# Comprehensive analysis of disulfidptosis-related genes and the immune microenvironment in heart failure

**DOI:** 10.3389/fcell.2024.1516898

**Published:** 2025-01-17

**Authors:** Linna Zhao, Juanjuan Zhang, Qiuhang Song, Cheng Dai, Yiping Qin, Aiying Li

**Affiliations:** ^1^ Department of Biochemistry and Molecular Biology, College of Basic Medicine, Hebei University of Chinese Medicine, Shijiazhuang, Hebei, China; ^2^ Faculty of Nursing, Hebei University of Chinese Medicine, Shijiazhuang, Hebei, China; ^3^ Hebei Key Laboratory of Chinese Medicine Research on Cardio-Cerebrovascular Disease, Shijiazhuang, Hebei, China

**Keywords:** heart failure, disulfidptosis, immune microenvironment, subtype, molecular clusters

## Abstract

**Background:**

Heart failure (HF) is a prevalent cardiovascular disease that currently lacks effective treatment options due to its intricate pathogenesis. A recent study has linked disulfidoptosis, a novel form of cell demise, with the development of a range of diseases. Nonetheless, the effect of disulfidoptosis on the immune microenvironment of HF is not well comprehended. In this paper, bioinformatics analysis was performed to investigate how disulfidptosis-related genes (DRGs) affect the immune microenvironment of HF.

**Methods:**

The expression of four DRGs was initially examined using bulk RNA-Seq and single-cell RNA sequencing data. A predictive model was subsequently developed. Consensus clustering was used to distinguish between the two clusters of DRGs. The effect of these DRGs on the characteristics of the immune microenvironment was further explored, such as infiltrating immune cells, immune response gene sets, and HLAs genes.

**Results:**

All four DRGs were dysregulated in HF samples. The predictive model based on these four DRGs effectively differentiated between HF patients and healthy individuals, which was validated in the experiment. These four DRGs were strongly associated with the abundance of infiltrating monocytes. Moreover, our analysis identified two distinct clusters of DRGs and these clusters exhibited differences in terms of immune cell abundance, immune response, and HLA gene expression. The biological functions associated with these differences were also revealed.

**Conclusion:**

Our discovery underscores the pivotal role of DRGs in shaping the diversity and intricacy of the immune microenvironment in HF.

## Introduction

Heart failure (HF) is an advanced stage of various heart-related conditions, primarily arising from structural changes or functional defects in the heart that impede ventricular filling and ejection. It is a major contributor to morbidity and mortality (Omar et al., 2023). In the first year of diagnosis, HF boasts a mortality rate of over 30%, placing substantial strain on healthcare systems worldwide due to its progressive nature and frequent hospital admissions (Batkai et al., 2021; Son et al., 2022).

The pathogenesis of HF is largely influenced by the abnormal activation of the immune system and the subsequent inflammatory response. Patients with HF frequently exhibit immune cell activation and inflammatory cell infiltration, resulting in inflammatory damage and cardiac tissue fibrosis. Additionally, this dysregulated activation of the immune system can trigger autoimmune reactions, characterized by self-attack on cardiomyocytes, ultimately worsening heart function (Van Bruggen et al., 2023; Cheng et al., 2023; Pu et al., 2023). Although considerable progress has been made in comprehending the pathogenesis and treatment of HF, the current situation remains worrisome. Therefore, an in-depth understanding of immunoregulation involved in HF may be key to unveiling its underlying pathological mechanisms and may provide new perspectives for innovative immunotherapies for HF.

Disulfidoptosis is a new mechanism of cellular death that manifests as cytoskeletal breakdown due to abnormal accumulation of intracellular disulfides (Liu X. et al., 2023). Research has unveiled that cells with elevated SLC7A11 expression, under conditions of glucose deficiency, experienced disulfidoptosis owing to abnormal accumulation of disulfide molecules. The excessive accumulation of disulfide molecules induces disulfide stress in actin cytoskeleton proteins and elevates disulfide bond levels in actin filaments. This, in turn, triggers filament contraction and cellular structural collapse, eventually leading to cell death.

Recent studies have demonstrated that disulfidoptosis is implicated in the underlying mechanisms of immunoregulation (Zhao Y. et al., 2023; Wang et al., 2023a; Xue et al., 2023). Wang et al. revealed a strong connection between disulfidoptosis-related gene (DRG) expression and M1 macrophages. Disulfidptosis may regulate the immune response in hepatocellular carcinoma by affecting the infiltration and activation status of immune cells (Wang et al., 2023a). Xue et al. have described the characteristics of long non-coding RNAs (lncRNAs) associated with disulfidptosis in the tumor immune microenvironment (IME). Their findings indicate that disulfidoptosis-related lncRNAs may be implicated in the infiltration and immune functionality of immune cells, thus potentially impacting the response to immunotherapy for tumors (Xue et al., 2023). Although there is growing evidence for the role of disulfidoptosis in immune responses, no studies have yet investigated its specific role in HF IME. Examining immune differences between samples from healthy individuals and HF patients, as well as among different HF subtypes, and studying how DRGs shift in response to these changes, may deepen our comprehension of HF mechanisms from a fresh perspective.

This study comprehensively assesses the regulatory patterns of DRGs in the context of HF. Our findings indicate that DRGs can effectively differentiate between samples from healthy individuals and HF patients and their expression patterns were validated in rats with HF induced by transverse aortic constriction (TAC). Furthermore, notable correlations were observed between the abundance of infiltrating immune cells and DRGs, implying a strong association between DRGs and immune regulatory processes. Samples from HF individuals were categorized into two distinct disulfidoptosis patterns based on the expression patterns of four DRGs. Notably, immune features were observed within these subtypes and the biological functions specific to each subtype were compared. Additionally, the expression of the four DRGs in the HF microenvironment was analyzed using the HF single-cell dataset. These collective findings underscore the substantial influence of DRGs on the IME of HF.

## Materials and methods

### Data preprocess

All data utilized in this research were from public datasets. Two bulk gene expression datasets [GSE57338 (136 normal samples, 177 HF samples) and GSE141910 (166 normal samples, 200 HF samples)] were downloaded from the Gene Expression Omnibus database (http://www.ncbi.nlm.nih.gov/geo/). The GSE57338 dataset (Liu et al., 2015) utilized the GPL11532 platform, specifically the Affymetrix Human Gene 1.1 ST Array with transcript (gene) version, while the GSE141910 dataset (Flam et al., 2022) was reliant on the GPL16791 platform, the Illumina HiSeq 2,500 (*Homo sapiens*). Both datasets were preprocessed using methods consistent with our prior study and were acquired using the R package “GEOquery” (Davis and Meltzer, 2007). Gene probes were annotated in the form of gene symbols. Probes lacking corresponding gene symbols and probes associated with multiple symbols were omitted. If duplicate gene symbols were found, the highest (maximum) value was selected. Recent publications provided four genes that were related to disulfidoptosis, namely, SLC7A11, SLC3A2, RPN1, and NCKAP1 (Zhao S. et al., 2023). These four genes were recognized as DRGs. Additionally, single-cell sequencing data were acquired from the GSE183852 dataset (Koenig et al., 2022), including 5 Transmural LV Apex samples of HF. Data were subjected to post-quality control, and the cell selection process was consistent with that outlined in the original study.

### Alteration analysis of DRGs between healthy and HF samples

The interplay of expression levels among four DRGs was assessed using Pearson correlation analysis across all samples and specifically within HF samples. To gauge expression differences in these DRGs between healthy and HF samples, the Wilcox test was applied. Additionally, the CIBERSORT (He et al., 2023) deconvolution algorithm (https://cibersort.stanford.edu/) was utilized to quantify the proportions of specific immune cell types based on the transcriptional data from the GSE141910 dataset. Multivariate logistic regression was employed to classify healthy and HF samples based on DRGs. To assess the discriminative capability of this signature, ROC (receiver operating characteristic) curve analysis was conducted. Additionally, the expression of particular genes was visualized using the featureplot function within the R Seurat package (Ren Q. et al., 2023).

### Identification of disulfidptosis characteristic pattern

An unsupervised cluster analysis was conducted on the expression of four DRGs utilizing the ConsensusClusterPlus package (Yu et al., 2023) to identify distinct clusters related to these DRGs. The K-Means algorithm and the euclidean distance metric were utilized, with resampling of 80% of the items and 1,000 replications (Ding and Jian, 2024). The optimal k value was chosen based on the proportion of ambiguous clustering. Principal components analysis (PCA) was performed and heatmaps were generated to further confirm the expression patterns of four DRGs in two characteristic patterns. Additionally, the abundance score of infiltrating immune cells, activity of immune checkpoints, and expression of histocompatibility leukocyte antigen (HLA) genes were compared between the two distinct characteristic patterns using the Wilcox test.

### Biological enrichment analysis for distinct disulfidptosis-related patterns

Gene set enrichment analysis (GSEA) was executed to identify the pivotal pathways and essential genes associated with different disulfidptosis patterns (Wang and Wang, 2023). The enrichment of pre-defined biological processes was assessed. The enriched pathways were sorted based on their normalized enrichment scores, and those with a significant level of P < 0.05 were selected for subsequent analyses. Gene set variation analysis (GSVA) is an unsupervised, non-parametric technique that assesses the variation in gene set enrichments within gene expression data. It is frequently utilized to examine fluctuations in pathways and biological processes among samples in an expression dataset. GSVA was leveraged in this study to explore disparities in biological processes between different disulfidptosis patterns (Wang Y. et al., 2023).

### Establishment of the TAC animal model

The TAC animal model and normal tissue samples used in this study were sourced from the laboratory research group. The rats in the TAC model group were anesthetized using an intraperitoneal injection of 0.3% pentobarbital sodium. Subsequently, the TAC surgery was performed using a 27-gauge needle, where a 6–0 silk suture was employed to ligate the aorta. In contrast, the sham group underwent an identical procedure, except for aortic ligation. After 4 weeks postoperatively, echocardiography was conducted to confirm the successful establishment of the TAC model. This research group had previously published articles utilizing these tissue samples (Zhou et al., 2022). In this study, these tissue samples were further used for the field of HF research.

### Western blot analysis

Proteins were extracted from rats using a lysis buffer consisting of RIPA buffer, phenylmethylsulfonyl fluoride, a protease inhibitor cocktail (from Roche, Switzerland), and phosphatase inhibitors (provided by Wuhan Servicebio, China). The protein content was quantified using a bicinchoninic acid kit, and proteins were subsequently separated through SDS-PAGE gel electrophoresis. Subsequently, the proteins were transferred onto a polyvinylidene fluoride membrane (from Millipore). The membrane was blocked with 5% nonfat dry milk in Tween/Tris-buffered saline at room temperature for 90 min. Following this, the membrane was incubated with primary antibodies overnight at 4°C. The employed antibodies encompassed anti-NPPA (rabbit polyclonal, 1:2,000, 27426-1-AP, Proteintech), anti-BNP (mouse polyclonal, 1:1,000, ab239510, Abcam), anti-beta MHC (rabbit polyclonal, 1:1,000, 22280-1-AP, Proteintech), anti-Collagen I (rabbit polyclonal, 1:1,000, 14695-1-AP, Proteintech), anti-Collagen III (rabbit polyclonal, 1:1,000, 22,734–1-AP, Proteintech), anti-α-SMA (rabbit polyclonal, 1:4,000, ab32575, Abcam), anti-SLC7A11 (rabbit polyclonal, 1:1,000, 26864-1-AP, Proteintech), anti-SLC3A2 (rabbit polyclonal, 1:10000, 15193-1-AP, Proteintech), anti-RPN1 (rabbit polyclonal, 1:1,000, 12894-1-AP, Proteintech), anti-NCKAP1 (rabbit polyclonal, 1:1,000, 12140-1-AP, Proteintech), and anti-GAPDH (mouse monoclonal, 1:10,000, 60,004–1-Ig, Proteintech). The following day, the membrane was incubated with the secondary antibody at room temperature for 90 min. Immunoreactive bands were detected utilizing the ECL chemiluminescent substrate kit and visualized using the chemiluminescent imager OmegaLum W (Minneapolis, MN, United States).

### Statistical analysis

Data calculations and statistical analyses were conducted utilizing R programming (https://www.r-project.org/, version 4.1.1). Pairwise differences were assessed using Wilcoxon tests, and statistical significance was denoted by P < 0.05 (*P < 0.05, **P < 0.01, ***P < 0.001, ****P < 0.0001, ns: not significant). Pearson correlation coefficients were computed for correlation analysis, and P < 0.05 indicated significance.

## Results

### The landscape of DRGs between healthy and HF samples

**FIGURE 1:**
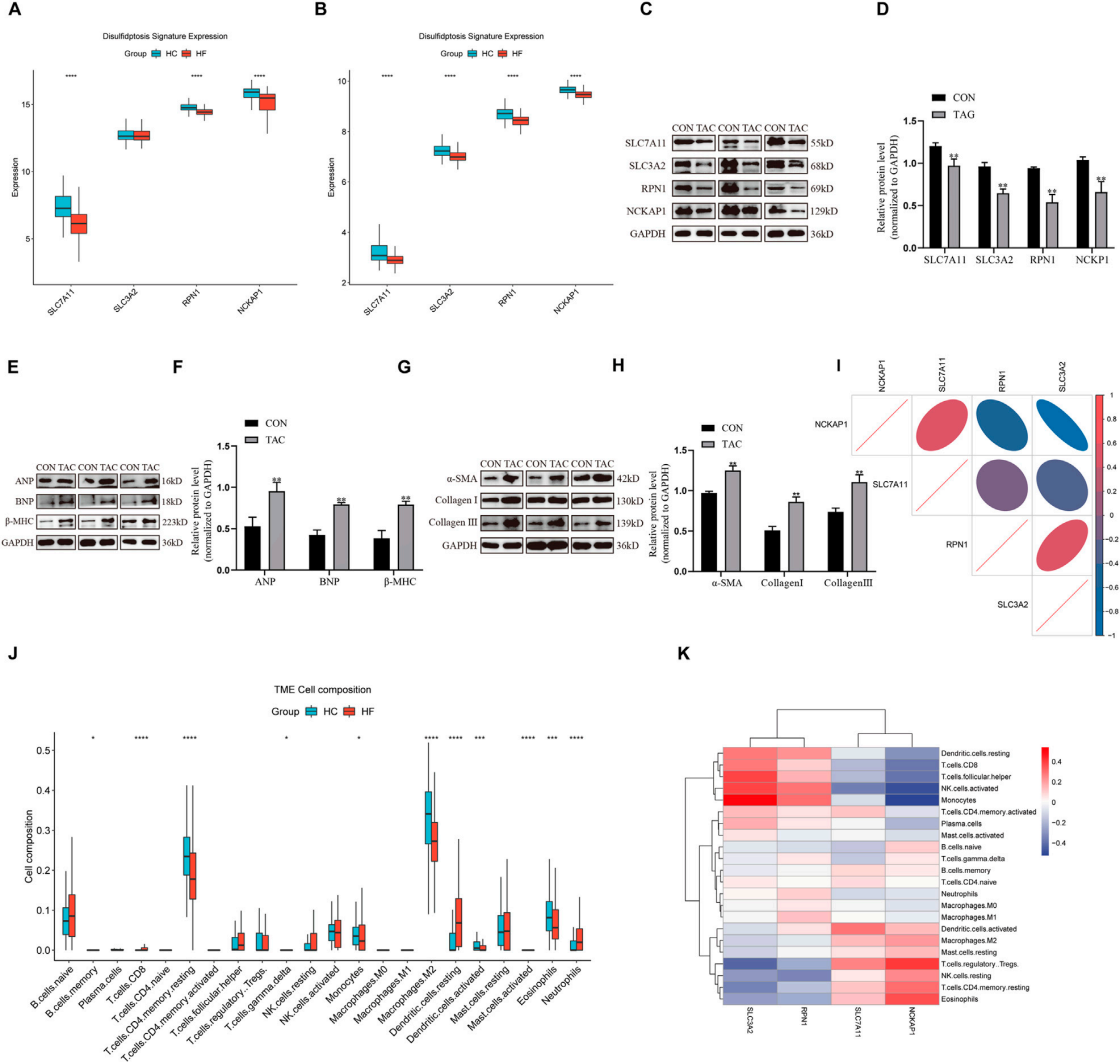
Expression landscape of DRGs in HF. **(A, B)** Boxplot of DRG expression levels in healthy samples and HF samples in both the GSE141910 and GSE57338 datasets. **(C, D)** Western blot analysis was performed to measure SLC7A11, SLC3A2, RPN1, and NCKAP1 expression levels in rats (n = 3). **(E–H)** Western blot analysis was performed to measure ANP, BNP, β-MHC, α-SMA, Collagen I, and Collagen III expression levels in rats (n = 3). **(I)** Correlations among the expression of 4 DRGs in HF samples. **(J)** Boxplots illustrating the differences in immune infiltration between HF and normal samples. **(K)** Correlation analysis of 4 differentially expressed DRGs and infiltrated immune cells. Western blotting data are shown as the mean ± SEM. Significance: *p < 0.05.

In the GSE141910 and GSE57338 datasets, the expression levels of four DRGs were significantly lower in the HF group than those in the healthy group (Figures 1A, B). Western blot demonstrated that in comparison to the CON group, the TAC group exhibited a notable decrease in the protein expression of SLC7A11, SLC3A2, RPN1, and NCKAP1 (Figures 1C, D). Further detection of protein levels of genes associated with myocardial hypertrophy and fibrosis showed significantly increased levels of ANP, BNP, β-MHC, Collagen I, Collagen III, and α-SMA. This suggested that the four DRGs might be related to this pathological change of HF (Figures 1E–H). Pearson correlation analysis showed that SLC7A11 expression was significantly positively correlated with NCKAP1 expression and negatively correlated with SLC3A2 expression; SLC3A2 expression was significantly positively correlated with RPN1 expression and negatively correlated with NCKAP1 expression; in addition, NCKAP1 expression was negatively correlated with RPN1 expression (Figure 1I). Differential analysis revealed notable distinctions in the abundance of certain infiltrating immune cells between HF samples and healthy samples, including B cells memory, T cells, monocytes, macrophages M2, dendritic cells, mast cells activated, eosinophils, and neutrophils (Figure 1J). The findings from correlation analysis suggested that the expression of DRGs exhibited strong connections with tumor-infiltrating immune cells. For instance, NCKAP1 and SLC3A2 were notably linked with monocytes, NK cells activated, and T cells regulatory (Tregs) (Figure 1K). These observations underscore the considerable variability in DRG expression between HF individuals and healthy subjects, indicating the possible roles of these atypical DRG expression patterns in HF.

### HF nomogram construction and verification

**FIGURE 2:**
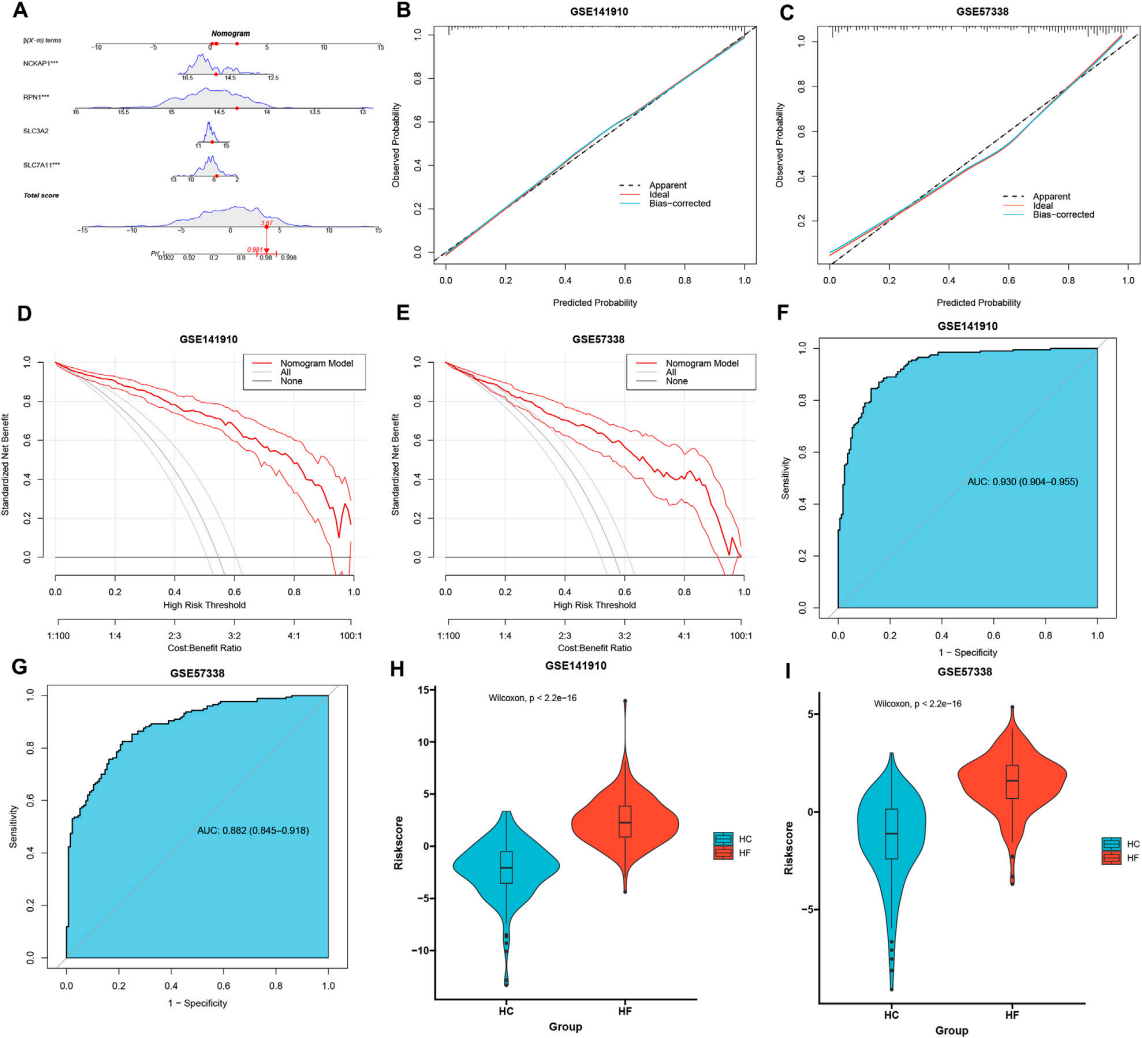
Construction and verification of a nomogram for HF. **(A)** Construction of a nomogram based on DRGs for prognostic signature. **(B, C)** Construction of the calibration curve for assessing the predictive efficiency of the nomogram in both the GSE141910 and GSE57338 datasets. **(D, E)** Decision curve analysis of risk prediction of the nomogram for HF in both the GSE141910 and GSE57338 datasets. **(F, G)** ROC curve validation of risk prediction of the nomogram for HF in both the GSE141910 and GSE57338 datasets. **(H, I)** The risk distribution between healthy and HF samples, where HF samples have a much higher risk score than healthy samples in both the GSE141910 and GSE57338 datasets.

The nomogram presented in Figure 2A was developed using the outcomes of multivariate logistic regression analysis to visualize the connection between four DRGs and the prognosis of HF. Calibration curves (Figures 2B, C) showed that the predicted curves closely approximated the ideal curve, indicating strong performance. The decision curve analysis further confirmed the enhanced clinical usefulness of the nomogram in predicting morbidity among HF patients (Figures 2D, E) in the training and validation sets. Furthermore, the area under the curve (AUC) was 0.930 (95% confidence interval (CI): 0.904–0.955) in the training set (Figure 2F) and 0.882 (95% CI: 0.845–0.918) in the validation set (Figure 2G). Notably, the risk score for disulfidptosis-related complications in HF samples was significantly higher than that in healthy samples in both the training (Figure 2H) and validation (Figure 2I) sets. These findings illustrate the effectiveness of the nomogram in differentiating between HF individuals and healthy people, underscoring the significant role of DRGs in HF development.

### Consensus clustering analysis of DRGs

**FIGURE 3:**
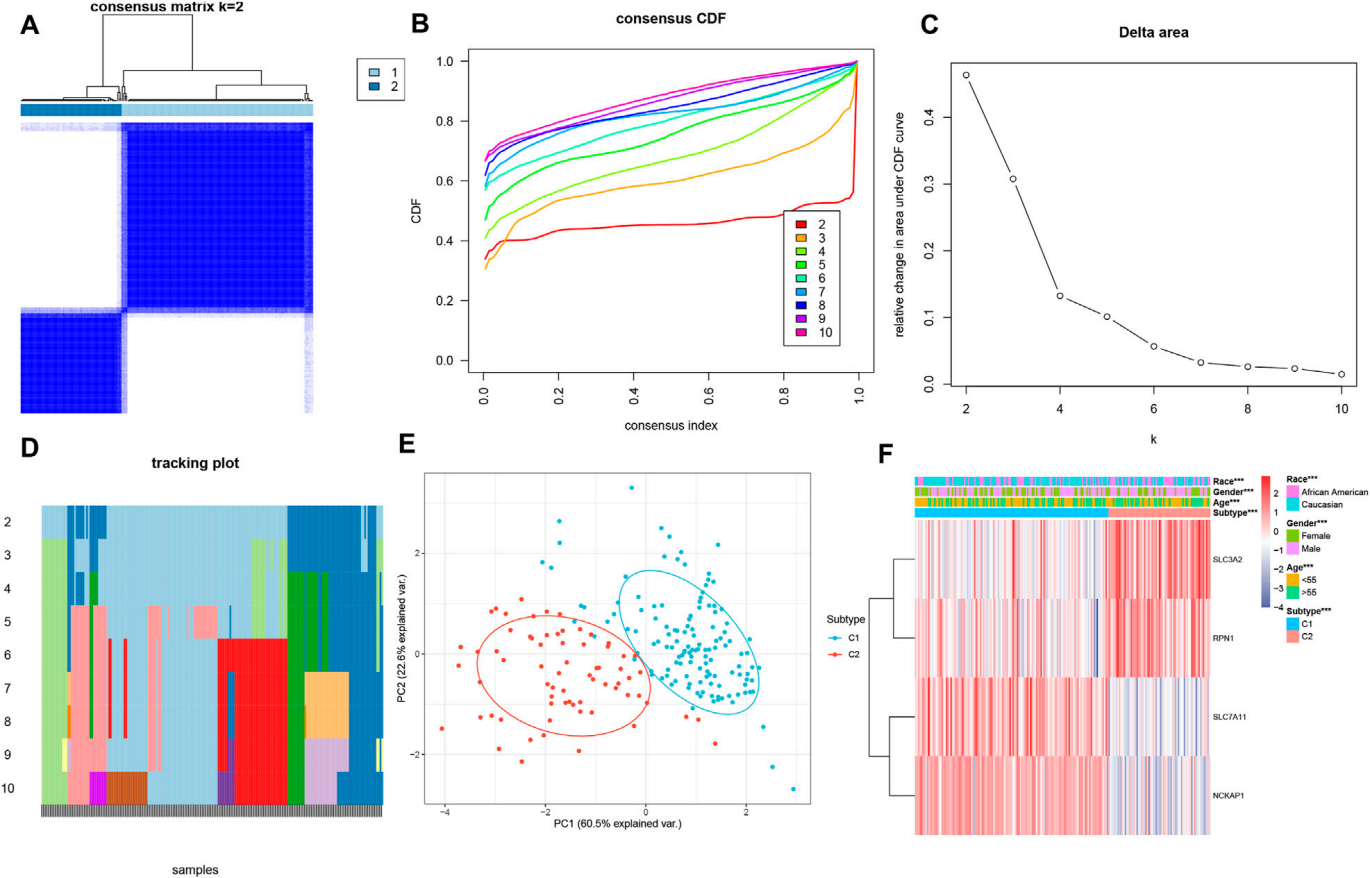
Consensus clustering analysis of DRGs. **(A)** Consensus clustering matrix when k = 2. **(B)** Consensus clustering cumulative distribution function (CDF) for k = 2–10. **(C)** Relative change in area under CDF curve for k = 2–10. **(D)** Tracking plot for validation of the clustering results. **(E)** PCA analysis showing significant differences between the two subtypes. **(F)** The heatmap illustrates the association of different clinical characters with the 2 subtypes.

The consensus clustering analysis indicated that the most stable grouping was achieved with k = 2 (Figures 3A–D). Subsequently, 200 HF samples obtained from the GEO database were categorized into two distinct groups C1 (n = 131) and C2 (n = 69). The PCA plot visually demonstrated distinct gene expression patterns between these two clusters (Figure 3E). Notably, distinctive DRG expression profiles were noted between the C1 and C2 groups (Figure 3F). Specifically, the expression levels of SLC7A11 and NCKAP1 were notably higher in the C1 group than those in the C2 group, whereas SLC3A2 and RPN1 exhibited higher expression levels in the C2 group than in the C1 group. The expression differences of these genes may reflect the differences between C1 and C2 patterns in these biological processes and may have certain implications for HF development and treatment. The visualization results of differences between the two groups in terms of age, sex, and race are listed in Supplementary Data 1.

### IME characteristics in distinct disulfidptosis patterns

**FIGURE 4:**
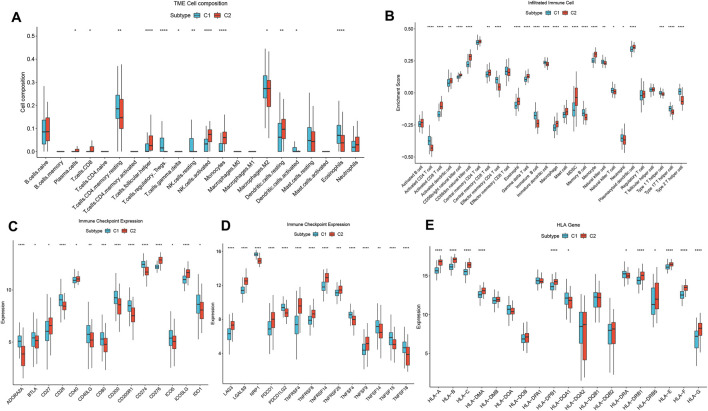
Diversity of immune characteristics among distinct disulfidptosis-related patterns. **(A)** The abundance differences of each immune infiltrating immunocyte in 2 disulfidptosis-related patterns. **(B)** The boxplot of 28 infiltrated immune cell types was calculated by GSEA. **(C, D)** The activity differences of each immune checkpoint in 2 disulfidptosis-related patterns. **(E)** The expression differences of each HLA gene in two disulfidptosis-related patterns.

Our analysis revealed significant differences in the abundance of immune cells between the two patterns (Figure 4A). Furthermore, immune infiltration analysis showed that the proportion of 28 types of immune cells was significantly different between the two patterns (Wilcoxon test, P < 0.05) (Figure 4B). Specifically, the C1 group exhibited higher levels of infiltrated Tregs, NK cells resting, macrophages M2, activated dendritic cells. The C2 group displayed enrichment in plasma cells, T cells CD8, T cells follicular helper, T cells gamma delta, activated NK cells, and monocytes, indicating a more robust profile of immune activation. Regarding immune checkpoints, the expression levels of ADORA2A, BTLA, CD28, CD40LG, CD80, CD200, and PDCD1LG2, were notably increased in the C1 group, while the expression levels of CD27, LAG3, and TNFRSF8, were significantly decreased compared to the C2 group (Figures 4C, D). Furthermore, the expression levels of HLA-A, HLA-B, HLA-C, HLA-DRB1, and HLA-E were notably elevated in the C2 group compared to the C1 group (Figure 4E), suggesting enhanced antigen presentation and immune recognition. Conversely, the C1 group exhibited increased expression of HLA-DQA1 and HLA-DRA, which may be linked to immunoregulation. These findings underscore substantial differences in the IME between the two disulfidptosis-associated patterns in HF, with C1 reflecting immune tolerance and C2 showing immune activation.

### Biological functional distinctions between DRG patterns

**FIGURE 5:**
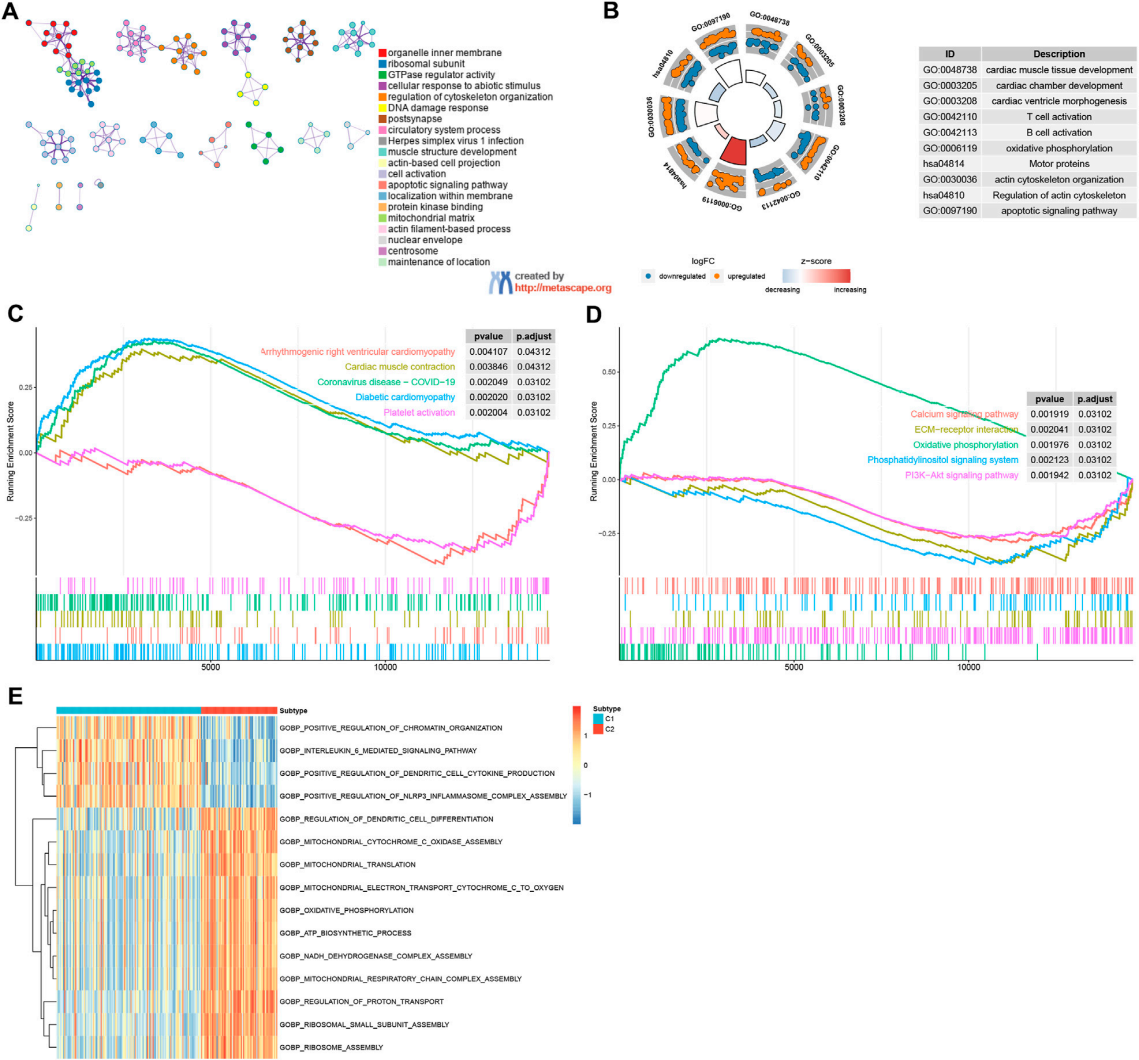
Enrichment analysis. **(A, B)** GO/KEGG functional enrichment analysis by Metascape. **(C, D)** GSEA functional and pathway enrichment analysis. **(E)** GSVA functional enrichment analysis.

GO/KEGG analysis confirmed that DRGs between C1 and C2 patterns were mostly involved in cardiac muscle tissue development, cardiac chamber development, T cell activation, B cell activation, motor proteins, actin cytoskeleton organization, regulation of actin cytoskeleton, and apoptotic signaling pathway (Figures 5A, B). The results from GSEA indicated that pathways such as cardiac muscle contraction, coronavirus disease − COVID−19, diabetic cardiomyopathy, and oxidative phosphorylation were enriched in the C2 group. Conversely, the C1 group exhibited enrichment in pathways including arrhythmogenic right ventricular cardiomyopathy, platelet activation, ECM−receptor interaction, phosphatidylinositol signaling system, calcium signaling pathway, and PI3K−Akt signaling pathway (Figures 5C, D). The GSVA analysis revealed several biology processes with distinct expression patterns, which were presented in a heatmap. In comparison to the C1 cluster, the GO biological process activities related to mitochondrial translation, mitochondrial electron transport, cytochrome c to oxygen, oxidative phosphorylation, ATP biosynthetic process, and NADH dehydrogenase complex assembly were more pronounced in the C2 cluster. Conversely, activities associated with the interleukin-6-mediated signaling pathway, positive regulation of dendritic cell cytokine production, and positive regulation of NLRP3 inflammasome complex assembly were more enriched in the C1 cluster (Figure 5E). These findings imply that the C1 cluster is linked to cardiovascular disease and inflammation, whereas C2 cluster is associated with energy metabolism and mitochondrial function. Furthermore, C1 and C2 patterns appear to play distinct roles in immunoregulation. The C1 subgroup is characterized by an enrichment of functional features related to immune-inflammatory processes, while the C2 subgroup may be more associated with immune cell metabolism and energy provision. These insights enhance our comprehension of the divergent roles played by C1 and C2 patterns in immunoregulation and offer valuable perspectives for further exploration of the immune system in HF pathogenesis.

### Single-cell analysis findings

**FIGURE 6:**
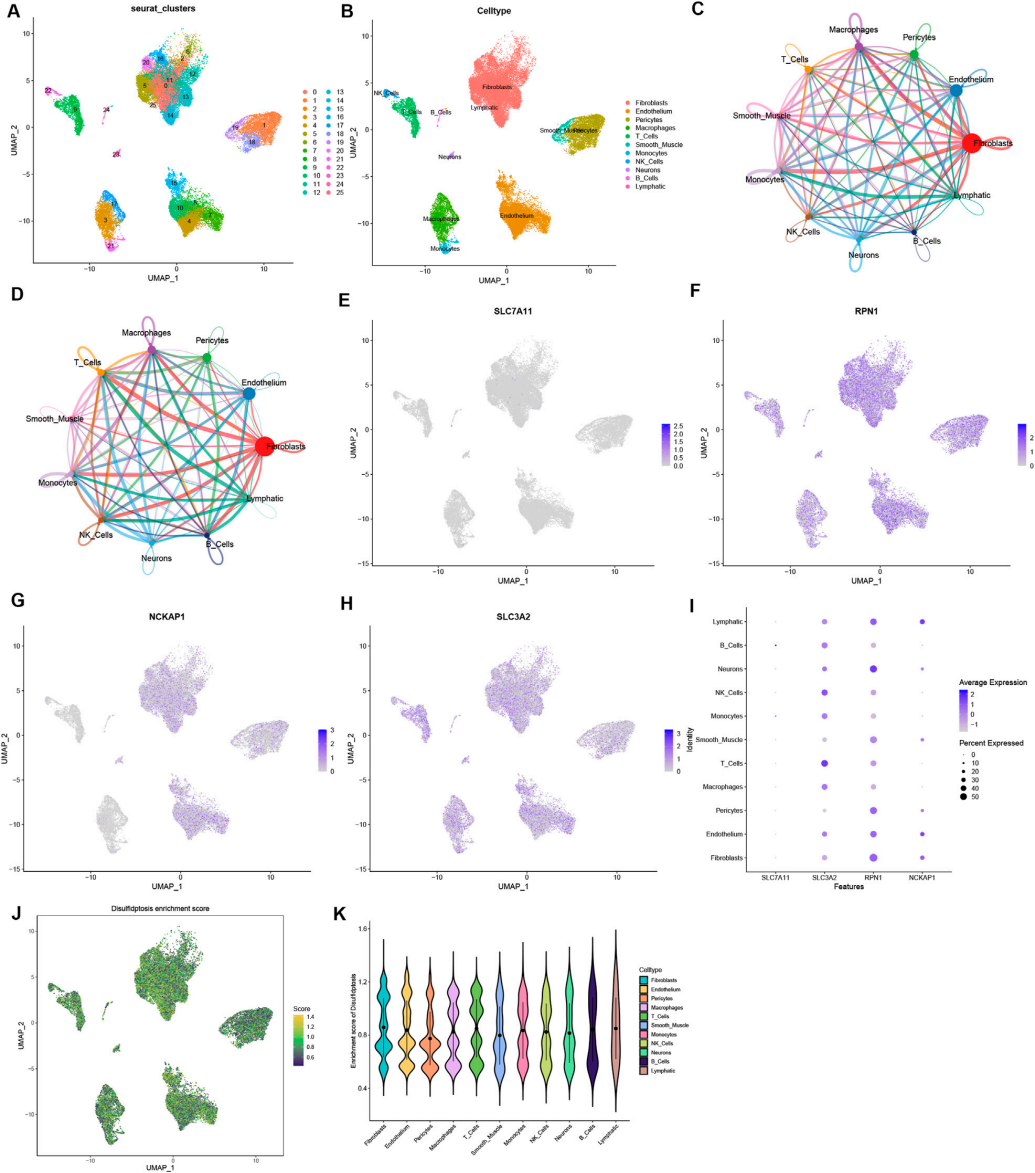
Single-cell sequencing data analysis. **(A)** Dimensionality reduction identified 15 principal components using the UMAP algorithm, and classification of 26 cell clusters. **(B)** Annotation of 26 cell clusters in HF by singleR. **(C, D)** Analysis of the number of interactions **(C)** and interaction strength **(D)** among different cell types in HF samples. **(E–H)** The expression of 4 DRGs in different types of cells. **(I)** The relationship between the expression level of DRGs and cell populations. **(J)** The disulfidptosis enrichment score (activity) in each cell. **(K)** The distribution of disulfidptosis score in different cell types.

The single-cell dataset GSE183852 was utilized for the analysis of the expression of four DRGs within the microenvironment of HF. Following quality control procedures, 35,715 single cells were obtained. The PCA using the top 2000 variable genes to reduce dimensionality identified 26 distinct cell populations (Supplementary Figure 2; Figure 6A). Subsequently, the cell identity for each cluster was assigned using a reference dataset from the human primary cell atlas, with 11 cell types defined (Figure 6B). These cell types included fibroblasts, endothelial cells, pericytes, macrophages, T cells, smooth muscle cells, monocytes, NK cells, neurons, B cells, and lymphocytes. Moreover, the intercellular communication networks from the scRNA-Seq data were analyzed using the CellChat package (Figures 6C, D). Many significant ligand–receptor pairs were found among the 11 cell types. Notably, SLC7A11 exhibited low expression across all cells, whereas RPN1 was expressed to some extent in all cell types (Figures 6E–H). Interestingly, SLC7A11 and SLC3A2 were mainly expressed in immune cells such as NK cells, T cells, and B cells, while RPN1 and NCKAP1 were highly expressed in fibroblasts, endothelial cells, and lymphocytes (Figure 6I). The ssGSEA function in the Seurat package was used to calculate the expression levels of 4 DRGs across all cells (Figure 6J). Of the 11 cell types, a higher disulfidptosis activity was observed in B cells and lymphocytes (Figure 6K). These findings imply that immune cells could notably impact the HF microenvironment. Specific DRGs are highly expressed in immune cells, which may be associated with HF development and immunoregulation.

## Discussion

HF is the most prevalent cardiovascular disease globally (McDonagh et al., 2021; Gui et al., 2023) and its complex pathogenesis primarily covers pathogens and immune responses (Adamo et al., 2021). Increasing evidence supports the crucial involvement of disulfidptosis in both innate and adaptive immune processes. To date, the role of disulfidptosis in immunity has been studied, particularly in infiltrating cells in the tumor microenvironment. These studies have reaffirmed its pivotal function in tumor immunity (Yang et al., 2023; Ren C. et al., 2023; Liu S. et al., 2023; Wang et al., 2023c). Hence, we anticipate that similar roles of disulfidptosis may present in the IME in HF. To comprehensively address these queries, we conducted a systematic investigation of disulfidptosis patterns in the IME of HF.

Through comprehensive analyses, a series of key insights were uncovered. Firstly, expression differences in four DRGs were found between healthy individuals and HF patients, indicating the involvement of DRGs in HF development. Secondly, the four DRGs were closely associated with immune characteristics, indicating the essential role of disulfidptosis in regulating the IME in HF. SLC7A11 is positively correlated with activated dendritic cells and plays an important role in the activation of dendritic cells, especially in regulating their metabolism and amino acid uptake (Liu J. et al., 2023). Some studies have also found the key role of SLC7A11 in the activation of dendritic cells by regulating oxidative stress and apoptosis (Gao Z. et al., 2023; Xia et al., 2023). Our analysis revealed that the abundance of infiltrating monocytes was positively correlated with SLC3A2 and negatively correlated with NCKAP1. Monocytes represent a vital component of the innate immune system and contribute significantly to maintaining homeostasis in HF (Ovchinnikov et al., 2023; Feng et al., 2023; Wang et al., 2023d). SLC3A2 expression in monocytes is intricately linked to its functionality (Xiang et al., 2023; Bajaj et al., 2016; Li et al., 2022), primarily the regulation of amino acid transportation. It interacts with other membrane proteins to form complexes that facilitate amino acid uptake and intracellular transport, which are crucial for proper monocyte functions and metabolism (Saha et al., 2022; Zhao et al., 2022). Additionally, SLC3A2 mediates the invasion and migration of monocytes (Huang et al., 2019) and collaborates with other membrane proteins to regulate the adhesion of monocytes to endothelial cells and various cell surfaces (Fuchs et al., 2018). The relationship between NCKAP1 and monocytes has not been clearly reported. However, the function of NCKAP1 in other immune cells has been studied. NCKAP1 can interact with cytoskeleton-associated proteins such as NCK and Wiskott-Aldrich syndrome protein to participate in cytoskeletal remodeling and cell motility. In monocytes, cytoskeletal remodeling and cell motility are also important processes, especially during monocyte migration and invasion (Noh et al., 2023; Zhang et al., 2020). Hence, it can be inferred that NCKAP1 might be involved in cytoskeletal reorganization and monocyte migration. Nevertheless, the specific function of NCKAP1 in monocytes and its relationship with monocytes still need to be clarified. Moreover, SLC3A2 also exhibits a robust association with T cells, a pivotal component of the immune system that is fundamental in the body’s defense mechanisms (Pellizzari et al., 2021; Kurihara et al., 2015; Bhuyan et al., 2014; Cantor et al., 2011). As a member of the solute carrier (SLC) family, SLC3A2 encodes CD98hc (CD98 heavy chain), a protein that functions as an amino acid transporter and is alternatively called the LAT1-CD98 complex (Zhou et al., 2023). This complex has important functions in T cells, especially in the metabolic regulation and immune response of T cells (Tian et al., 2023). The growth, proliferation, activation, and immune response of T cells all depend on an adequate supply of amino acids. When T cells are stimulated externally and then proliferate and differentiate rapidly, their metabolic demands increase significantly. SLC3A2 provides essential energy and substances for T cells by mediating the uptake of amino acids (Cibrian et al., 2016). However, the specific mechanism of SLC3A2 and its relationship with different types of T cell subpopulations require further investigation.

These findings may indicate the immunoregulation mechanism of disulfidptosis in HF. The classifier, built upon four DRGs, effectively discerned healthy and HF samples, reinforcing the significant role of DRGs in HF. Moreover, the consensus clustering analysis of HF samples based on the expression profiles of these four DRGs identified two distinct subtypes with unique disulfidptosis patterns and immune features. The C1 subgroup exhibited increased populations of resting CD4 memory T cells, Tregs, and M2 macrophages. These cell types are essential in maintaining immune tolerance and tissue homeostasis. CD4 memory T cells contribute to long-term immune responses by preventing unnecessary activation, thereby mitigating tissue damage during HF (Bansal et al., 2017). M2 macrophages exert regenerative roles by participating in tissue repair and anti-inflammation, although their immunosuppressive properties reduce pathogen clearance, posing a risk for infections in vulnerable HF patients (Wang et al., 2023e). In contrast, the C2 subgroup exhibited a robust activation profile, with increased populations of CD8 T cells, activated NK cells, and plasma cells. CD8 T cells are crucial for recognizing and eliminating infected or damaged cardiomyocytes, but their heightened activity in this context may exacerbate inflammation and tissue injury. Activated NK cells play a dual role in combating infections. However, their uncontrolled activation can cause collateral damage to cardiac tissues. The abundance of plasma cells indicates an aggressive humoral immune response, which produces antibodies to combat infections but could also contribute to autoimmunity, thus complicating HF progression (Schmidt et al., 2023; Bai et al., 2023). Evaluating the immune signatures of each subtype solidified the credibility of our immunophenotype classification based on different DRGs. This approach for immune subtypes may enhance our comprehension of the underlying immunoregulation mechanisms for the application of precise therapeutic strategies. It allows for detailed categorization of HF at the phenotypic, molecular, and immune levels (Zhang et al., 2020). The molecular subtyping strategy is common in the field of oncology, and new molecular subtypes may refine treatment protocols (Li et al., 2019; Teo et al., 2019). Therefore, two distinct disulfidptosis patterns in HF signify that the disulfidptosis pattern in myocardial tissue can be considered as an alternative pathobiology-based classification for HF, one that correlates with the disease’s phenotypic characteristics. Furthermore, single-cell analysis found multiple immune cell types in the HF microenvironment, and DRGs played an important role in these immune cell types. RPN1 is highly expressed in macrophages, T cells, and fibroblasts and is involved in protein degradation and endoplasmic reticulum stress response. Low RPN1 expression in HF may lead to the polarization of macrophages toward a pro-inflammatory phenotype, enhancing inflammatory responses and exacerbating cardiac injury. In addition, low expression of RPN1 may also be associated with endothelial cell dysfunction, which in turn increases microvascular permeability and further promotes the onset and progression of cardiac inflammation (Zong et al., 2024; Pan et al., 2024). High NCKAP1 expression in fibroblasts and lymphocytes is associated with cell migration and functional regulation. In HF, low NCKAP1 expression may impair fibroblast function, thus contributing to their overactivation and fibrosis. For lymphocytes, NCKAP1 deletion may lead to decreased localization and activation, which reduces immune monitoring and response to cardiac pathology, thus contributing to immune tolerance and further aggravating the progression of HF (Steffen et al., 2004; Whitelaw et al., 2020; Yamamoto et al., 2001). SLC3A2, as an amino acid transporter protein, is expressed predominantly in macrophages and T cells. Its low expression is closely associated with metabolic dysregulation in macrophages, which may lead to impaired function and enhanced pro-inflammatory responses (Xiong et al., 2024; Zhao et al., 2024). In HF, SLC3A2 deficiency may affect the acquisition and metabolism of amino acids, limit the ability of macrophages to adapt to the microenvironment, and consequently exacerbate inflammatory injury in the heart. These findings provide important clues to our understanding of the pathogenesis of HF and the role of disulfidptosis and immunity in HF.

Our study represents the initial systematic analysis of the connection between DRGs and the IME of HF. It has achieved fruitful results and opened up a fresh perspective for research on the immune-related pathogenesis of HF with a focus on disulfidptosis. We initially introduce the latest disulfidptosis mechanism in HF and establish the implication of DRGs in the regulation of the IME in HF. By integrating the latest understanding of disulfidptosis mechanisms into the IME, this study unveils the novel pathogenesis of HF. This pioneering research has made a significant contribution to bridging the knowledge gap in the field of disulfidptosis in HF. This study is expected to inspire numerous researchers to embark on disulfidptosis-related investigations in the context of HF and provide valuable guidance based on the myriad results presented. However, we must admit that this study has certain limitations. First, our current research primarily relies on comprehensive bioinformatics analysis. It lacks additional support from experimental and clinical validation. Therefore, further confirmation through experimental and clinical findings is required. Nevertheless, given the robust outcomes observed in numerous oncology studies based on TCGA data analysis, we believe that the results derived from our bioinformatics analysis are reliable. Second, common analytical methods are utilized to estimate the quantity of immune cells, but single-cell sequencing is required to determine the count of immune cells most accurately. Additionally, more comprehensive clinical data are essential to validate the predictive performance of the model, and additional external validation cohorts are imperative to ensure the robustness of the diagnostic model (Gao Y. et al., 2023; Weng et al., 2023; Ye et al., 2023). Nonetheless, all of our findings undeniably affirm the substantial influence of DRGs on the immune signatures in HF. These findings yield fresh perspectives for comprehending the pathogenesis of HF.

## Conclusions

Our investigation unveils the inherent regulatory mechanisms of DRGs within the IME of HF. Disulfidptosis exerts a significant influence on the diversity and intricacy of the IME. This comprehensive exploration of HF and disulfidptosis enhances our comprehension of the underlying immune regulatory network in HF and may stimulate the development of more effective therapeutic approaches.

## Data Availability

The original contributions presented in the study are included in the article/Supplementary Material, further inquiries can be directed to the corresponding author.
