# Supplementary figures and images for: Comprehensive analysis of disulfidptosis-related genes and the immune microenvironment in heart failure
Source: Front Cell Dev Biol. 2025 Jan 17;12:1516898. doi: 10.3389/fcell.2024.1516898 (PMC11782221; doi:10.3389/fcell.2024.1516898)

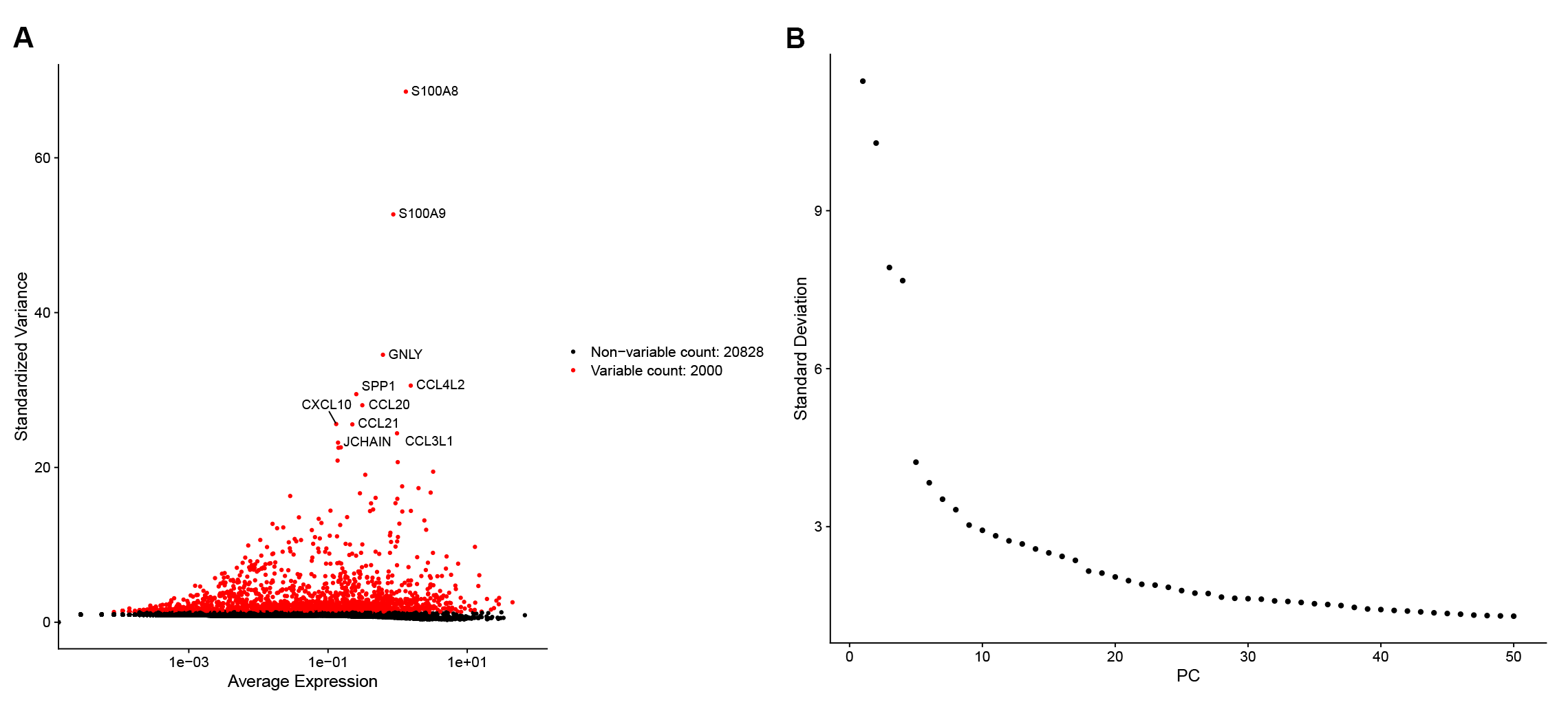

Supplement: Supplementary file 1 [file Image3.tif]

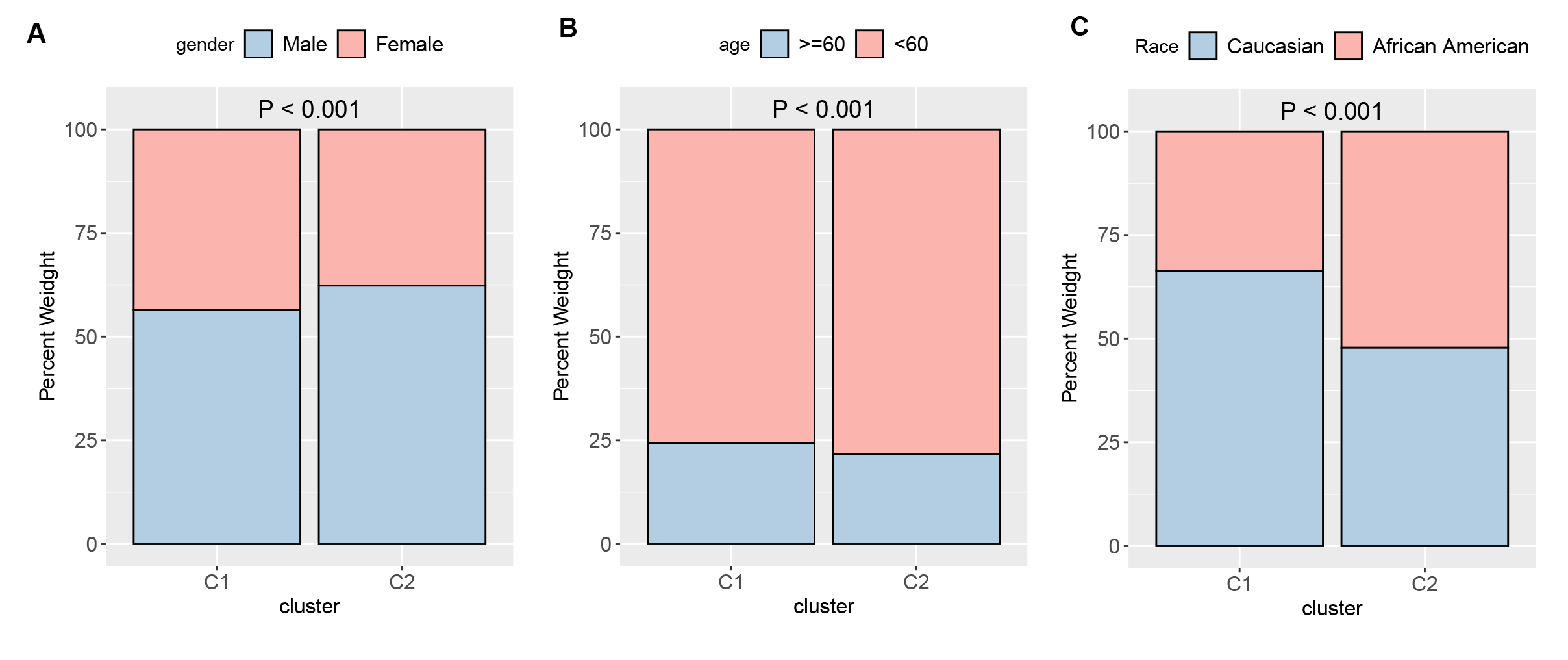

Supplement: Supplementary file 2 [file Image2.tif]

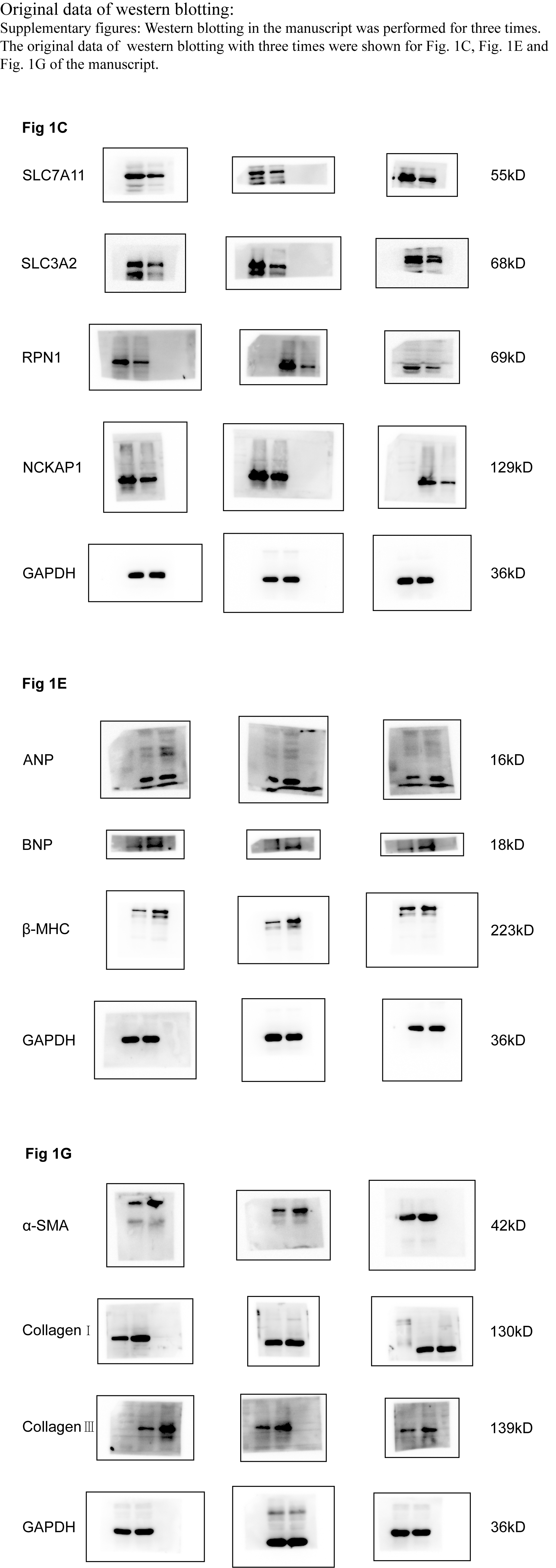

Supplement: Supplementary file 3 [file Image1.tif]
